# Lysosphingolipids in ceramide-deficient skin lipid models

**DOI:** 10.1016/j.jlr.2024.100722

**Published:** 2024-12-07

**Authors:** Georgios Paraskevopoulos, Lukáš Opálka, Andrej Kováčik, Anna Paraskevopoulou, Eleni Panoutsopoulou, Irene Sagrafena, Petra Pullmannová, Robert Čáp, Kateřina Vávrová

**Affiliations:** 1 Skin Barrier Research Group, Charles University, Faculty of Pharmacy in Hradec Králové, Hradec Králové, Czech Republic; 2 Plastic Surgery Clinic, Sanatorium Sanus, Hradec Králové, Czech Republic

**Keywords:** skin barrier, permeability, ceramide, lysolipid, glucosylsphingosine, sphingosine-phosphorylcholine, fatty acid, lipid model

## Abstract

Ceramides are key components of the skin's permeability barrier. In atopic dermatitis, pathological hydrolysis of ceramide precursors - glucosylceramides and sphingomyelin - into lysosphingolipids, specifically glucosylsphingosine (GS) and sphingosine-phosphorylcholine (SPC), and free fatty acids (FFAs) has been proposed to contribute to impaired skin barrier function. This study investigated whether replacing ceramides with lysosphingolipids and FFAs in skin lipid barrier models would exacerbate barrier dysfunction. When applied topically to human stratum corneum sheets, SPC and GS increased water loss, decreased electrical impedance, and slightly disordered lipid chains. In lipid models containing isolated human stratum corneum ceramides, reducing ceramides by ≥ 30% significantly increased permeability to four markers, likely due to loss of long-periodicity phase (LPP) lamellae and phase separation within the lipid matrix, as revealed by X-ray diffraction and infrared spectroscopy. However, when the missing ceramides were replaced by lysosphingolipids and FFAs, no further increase in permeability was observed. Conversely, these molecules partially mitigated the negative effects of ceramide deficiency, particularly with 5%–10% SPC, which reduced permeability even compared to control with “healthy” lipid composition. These findings suggest that while ceramide deficiency is a key factor in skin barrier dysfunction, the presence of lysosphingolipids and FFAs does not aggravate lipid structural or functional damage, but may provide partial compensation, raising further questions about the behavior of lyso(sphingo)lipids in rigid multilamellar lipid environments, such as the stratum corneum, that warrant further investigation.

The stratum corneum (SC), the outermost layer of the skin, is the body's first line of defense against the environment, including protection against water loss and external threats such as harmful compounds and bacteria (1). For competent SC barrier function, the unique structure and composition of the SC extracellular lipid matrix is critical (2, 3). This lipid matrix is devoid of phospholipids and consists mainly of ceramides (Cers), free fatty acids (FFAs), and cholesterol (Chol) in an approximately equimolar ratio, with minor components such as cholesteryl sulfate (CholS) (4). These lipids form multiple minimally hydrated lamellae between terminally differentiated corneocytes. The major barrier components, Cers (approximately 50% by weight of the lipid matrix), are highly lipophilic molecules (5, 6, 7) and are stored in the form of their more polar precursors, sphingomyelins (SMs) or glucosylceramides (GlcCers), before release into the extracellular space of the SC (8). Once released into the SC, GlcCer is the source of all Cer subclasses through the action of glucocerebrosidase (9); some Cer subclasses are also formed from SMs by sphingomyelinase (Fig. 1) (10).

**Fig. 1:**
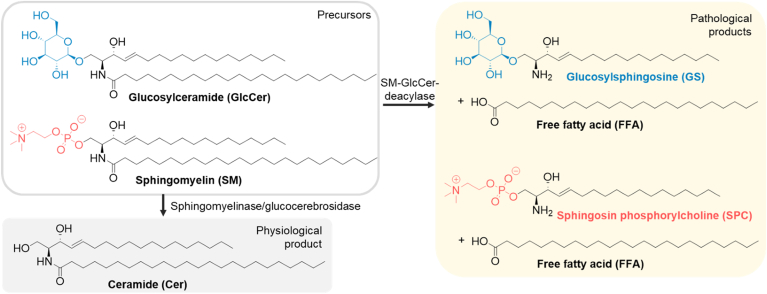
Schematic of the release of barrier Cers from their precursors GlcCers and SMs and the pathological pathway of degradation of these precursors by SM-GlcCer-deacylase to the lysosphingolipids GS or SPC and FFAs.

Alterations in the Cer-generating pathways have been implicated in several skin diseases such as atopic dermatitis (11). One such change described in atopic dermatitis is the pathological expression of sphingomyelin-glucosylceramide deacylase (SM-GlcCer-deacylase), an enzyme that hydrolyzes the amide bond of SMs and GlcCers to release a free fatty acid and a lysosphingolipid, either sphingosine-phosphorylcholine (SPC) from SM or glucosylsphingosine (GS) from GlcCer (Fig. 1) (12, 13). This aberrant pathway leads to an approximately threefold increase in lysosphingolipid levels in the SC (14) and has been suggested as a causative factor for Cer deficiency in atopic dermatitis (15). In addition to atopic dermatitis (16), lysosphingolipids have been implicated in several diseases (17), such as SPC in cancer (18, 19) and cardiovascular diseases (20) and GS in Gaucher disease (21).

It seems intuitive that lysosphingolipid with FFA cannot replace the missing Cer in the skin barrier and that this modification in skin lipid composition would indeed be responsible for the changes in skin microstructure and increased permeability observed in atopic dermatitis patients. For example, a related lipid class, lysophospholipids, destabilizes and permeabilizes membranes, a property that has also been investigated for potential use in drug delivery (22, 23). For example, lysophosphatidylcholines and lysophosphatidylethanolamines affect the spontaneous curvature and bending of dioleoylphosphatidylethanolamine membranes (24). Monopalmitoylphosphatidylcholine and monostearoylphosphatidylcholine increase the permeability of dipalmitoylphosphatidylcholine liposomes to dithionite ion and the release of doxorubicin (25). Similar liposomes with monopalmitoylphosphatidylcholine completely released their contents under near-infrared irradiation (26). Lysophosphatidylcholines and FFAs (separately or in combination) were further investigated for doxorubicin release from liposomal formulations (27). However, for GS or SPC within the skin lipid barrier, such data are currently unavailable, and comparing the behavior of structurally distinct lipids across systems with differing architectures, such as vesicles and multilayers with variations in curvature, is not feasible.

Thus, despite these indications and the clearly different physicochemical properties of GS or SPC and Cers, it is not possible to unequivocally link these changes resulting from the hydrolysis of GlcCers and SMs (formation of lysosphingolipids and FFAs at the expense of Cers) to the increased permeability of the skin barrier in atopic dermatitis. This is because several other alterations in lipid metabolism have been identified in this disease (eg insufficient elongation of acyl chains (28)) and their relative contribution to the overall permeability barrier function is unknown.

Therefore, this study aimed to describe the basic aspects of the effects of GS and SPC on skin barrier lipids. First, we wanted to determine whether topically applied lysosphingolipids on isolated human SC would affect its functional characteristics, water loss and electrical impedance. Also, lipid chain order was examined by infrared spectroscopy. For a more detailed investigation, we prepared lipid films composed of a Cer fraction isolated from human SC, FFAs, Chol, and CholS as models of a normal/healthy barrier (control) and gradually replaced 5 mol% - 100 mol% Cer fraction with GS + FFAs or SPC + FFAs. Models with appropriately reduced levels of Cers without lysolipid + FFA replacement were studied as controls. We monitored the permeability of these models to two model compounds with different physicochemical properties, water loss, and electrical impedance. Lamellar phases were investigated by X-ray diffraction (XRD) and lipid chain order and packing by Fourier transform infrared spectroscopy.

## Materials and methods

### Chemicals

SPC, GS, SM, and Cers (used as analytical standards: NS [d18:1/24:0], NP [t18:0/24:0], AS [d18:1/h24:0] and AP [t18:0/h24:0]) were purchased from Avanti Polar Lipids (Alabaster, AL, USA). Cers EOS [d18:1/h32:0/18:2] and EOP [t18:0/h32:0/18:2] used as analytical standards were synthesized according to Opalka *et al*. (29). Cholesterol from lanolin, sodium cholesteryl sulfate (CholS), hexadecanoic acid (C_16_), octadecanoic acid (C_18_), eicosanoic acid (C_20_), docosanoic acid (C_22_), tetracosanoic acid (lignoceric acid, C_24_), theophylline anhydrous (TH), indomethacin (IND), gentamicin sulfate from Micromonospora purpurea (700 U/mg), 1,2-propanediol (propylene glycol), trypsin from porcine pancreas (1500 U/mg), salts for buffer preparation and solvents of HPLC grade were purchased from Merck. Water was deionized, distilled, and filtered through a Millipore Q purification system.

### Human skin, isolation of SC, and Cer fraction

The skin was obtained from female individuals who had undergone abdominal plastic surgery and had given their prior written informed consent. The procedure was approved by the Ethics Committee of the Sanus First Private Surgical Centre (No. 221103), and conducted according to the principles of the Declaration of Helsinki. The subcutaneous fat layer was removed from the skin samples with a scalpel, and the skin was washed with water, dried, and then stored at −20°C before use.

The frozen skin was gently thawed and immersed in Millipore water at 60°C for 2 min. The epidermis was gently removed with tweezers. The epidermis was then incubated overnight with 0.5% trypsin in PBS solution (pH = 7.4) at 32°C (30). The isolated SC was briefly washed with PBS, acetone—to remove skin surface lipids and contaminants—and Millipore water.

Lipids were extracted from the SC sheets with a series of chloroform: methanol mixtures (2:1, 1:1, and 1:2 v/v for 2 h each) (31). The combined organic solutions were evaporated under reduced pressure and the Cer fraction was isolated by column chromatography with a gradient of chloroform to chloroform/methanol 1:1 v/v (32). The physiological composition of Cers was verified using high-performance thin-layer chromatography (HPTLC) (33, 34).

### SC lipid models

Control lipid models were prepared from the isolated human SC Cers with FFAs and Chol in a 1:1:1 M ratio with 5 wt% CholS. The FFA mixture was composed of palmitic acid 1.3%, stearic acid 3.3%, arachidic acid 6.9%, behenic acid 47.1%, and lignoceric acid 41.4% (35, 36). This lipid composition roughly simulates the native SC extracellular lipid matrix. To mimic the hydrolysis of Cer precursors to a lysosphingolipid and FFA, and a concomitant Cer deficiency in the SC, in the other models 5, 10, 20, 30, 50, and 100 mol% of the Cer fraction was replaced by an equimolar mixture of SPC + FFAs or GS + FFAs. Models with appropriately reduced levels of Cers without lysolipid + FFA replacement were studied as controls.

To prepare the models, FFAs, Chol, and Cers were each dissolved in 2:1 hexane/96% ethanol (v/v) (note: human Cers produced a fine suspension that was carefully homogenized before use), and CholS was dissolved in 96% ethanol. The organic solutions were mixed in the desired proportions, evaporated under a stream of nitrogen, and vacuum-dried over solid paraffin and phosphorus pentoxide for 24 h. Nuclepore track-etched polycarbonate filters of 0.015 μm pore size (Whatman, Kent, Maidstone, United Kingdom) were washed in 2:1 hexane/96% ethanol (v/v), dried, and mounted in steel holders. Lipids were dissolved in 2:1 hexane/96% ethanol (v/v) at 4.5 mg/ml and sprayed onto the filters (3 × 100 μl/cm^2^) under a nitrogen stream using a Linomat 5 (Camag) equipped with an additional module for y-axis movement. The lipid films were heated to 90°C (above their phase transition temperature) for 10 min and slowly (over 3 h) cooled to ambient temperature. During this process, the lamellar structure was formed.

### Permeability of lipid models and SC sheets

Model lipid films on polycarbonate filters were sandwiched between Teflon holders with an available diffusion area of 0.5 cm^2^ and mounted in Franz diffusion cells. The acceptor compartment was filled with PBS at pH 7.4 containing 50 mg/l gentamicin. The precise volume of the buffer (7.0 ± 1.0 ml) was individually measured for each cell and was included in the flux calculation. The acceptor phase was stirred at 32°C throughout the experiment. After a 12 h equilibration at 32°C, the transepidermal water loss (TEWL) was measured, followed by the measurement of electrical impedance (see below). Then, 100 μl of either 5% theophylline (TH) or 2% indomethacin (IND) in 60% propylene glycol were applied to the lipid films. Note: 60% propylene glycol has no adverse effects on the membranes (37, 38). The donor samples were saturated with the pertinent model drug to maintain the same thermodynamic activity throughout the experiment. This setup ensured sink conditions for the model permeants. Samples of the acceptor phase (300 μl) were withdrawn every 2 h over 10 h and were replaced by the same volume of fresh PBS. During these periods, steady-state conditions were reached. The cumulative amounts of TH and IND that penetrated through the lipid membrane were analyzed by HPLC (see below), were corrected for the acceptor phase replacement and exact acceptor volume of the diffusion cells, and were plotted against time. The steady-state flux of TH or IND [μg∗cm^−2^∗h^−1^] was calculated as the slope of the linear regression function obtained by fitting the linear region of the plot in Microsoft Excel.

The permeability of the lysosphingolipid-treated SC sheets was examined using a similar procedure. The SC sheets (supported on 0.45 μm polycarbonate filters from Whatman, Kent, Maidstone, United Kingdom) were sandwiched between Teflon holders with an available diffusion area of 1.0 cm^2^ and mounted in Franz diffusion cells. After filling the acceptor with PBS and equilibration, TEWL was measured to ensure the SC integrity. 100 μl of a 1% solution of SPC or GS in propylene glycol/ethanol (7:3, v/v) was applied to each SC, control samples received the same volume of solvent without the lysosphingolipids. After 12 h, the samples were removed, the SC surface was carefully cleaned with Q-tips cotton swabs and the SC samples were allowed to equilibrate overnight without occlusion. TEWL was then measured, the Franz cells disassembled and the SC sheets were analyzed by infrared spectroscopy and HPTLC for the determination of the lysosphingolipid content (see below).

### High-performance thin-layer chromatography (HPTLC)

HPTLC was used to verify the composition of the isolated human Cers and to determine the lysosphingolipids in the SC sheets using silica gel 60 HPTLC plates (10 × 10 cm, Merck, Darmstadt, Germany). The treated area of the SC sheet was cut and extracted with a series of chloroform/methanol mixtures (2:1, 1:1 and 1:2, v/v, for 2 h each) at room temperature. After solvent evaporation, the extracted SC lipids (or Cer fraction) were dissolved in 100 μl of 2:1 chloroform/methanol and 10–30 μl of each lipid sample was sprayed onto the plate using a Linomat 5 (Camag). Standard lysosphingolipids, Cers, Chol and lignoceric acid were each dissolved in chloroform/methanol 2:1 (v/v), CholS, GlcCer, SM and phospholipids were each dissolved in chloroform/methanol 1:1 (v/v) at 1 mg/ml and mixed to construct the calibration curve. Chol, FFAs, and Cers were separated using 190:9:1.5 chloroform/methanol/acetic acid (v/v/v) as the mobile phase, polar lipids were separated using chloroform/methanol/acetic acid/H_2_O 66:25:6:3 (v/v/v/v) in an ADC2 chamber (Camag). Lipids were visualized using derivatization reagent (7.5% CuSO_4_, 8% H_3_PO_4_, and 10% methanol in water) and heating at 160°C and were quantified by densitometry using a TLC Scanner 3 and visionCATS software (Camag).

### High-performance liquid chromatography (HPLC)

The model permeants (TH and IND) in the acceptor phase samples were analyzed using a Shimadzu Prominence instrument (Kyoto) on a LiChroCART 250-4 column (LiChrospher 100 RP-18, 5 μm, Merck) using isocratic elution. The mobile phases consisted of methanol/0.1 M NaH_2_PO_4_ in a 4:6 (v/v) ratio at 35°C and a flow rate of 1.2 ml/min for TH and of acetonitrile/water/acetic acid in a 90:60:5 (v/v/v) ratio at 40°C and a flow rate of 2 ml/min for IND. The detection wavelengths for TH and IND were 272 and 260 nm, respectively. Both methods have been previously validated (38).

### Water loss

Water loss through the lipid models and SC sheets was measured in the Franz cells with their upper parts removed using an AquaFlux AF 200 instrument (Biox Systems Ltd, Stansted, United Kingdom) at 30%–36% relative air humidity and 24–26°C external temperature (the acceptor phase temperature was maintained at 32°C). The Aqua-Flux uses the condenser chamber method of measurement. The measured TEWL value is defined as the steady-state flux density of water diffusing through the SC or lipid film.

### Electrical impedance

The electrical impedance was measured using a 4080 LCR meter (Conrad Electronic, Hirschau, Germany) at an alternating frequency of 120 Hz. The measuring range of the instrument was 20 Ω to 10 MΩ, with an error at kΩ values less than 0.5%. 500 μl of phosphate-buffered saline was applied to the donor compartment of the Franz diffusion cell, and the impedance (in kΩ∗cm^2^) was measured using stainless steel probes placed in the donor and acceptor compartments. At the end of the measurements, the buffer was carefully removed by swabs.

### X-ray diffraction (XRD)

The lipid mixtures were prepared in the same manner as for the permeation experiments, were sprayed onto a cover glass (22 × 22 mm), and annealed as described above. The XRD data were collected at ambient temperature (25°C) with an X'Pert PRO θ-θ powder diffractometer (PANalytical B.V., Almelo, Netherlands) using the parafocusing Bragg-Brentano geometry with Co Kα radiation (λ = 1.790307 Å, U = 35 kV, I = 40 mA) in modified sample holders over the angular range of 0.6–30° [2θ]. The data were scanned with an X'Celerator ultrafast detector with a step size of 0.0167° [2θ] and a counting time of 20.32 s∗step^−1^. The data were evaluated using the HighScore Plus software package (PANalytical B.V., Almelo, Netherlands). The XRD diffractograms show the scattered intensity as a function of the scattering vector Q [nm^−1^], which is proportional to the scattering angle 2θ according to the equation: Q = 4π sinθ/λ (λ = 0.1790307 nm is the wavelength of the X-rays). The repeat distance *d* [nm] characterizes the regular spacing of parallel lipid bilayers arranged on a one-dimensional lattice, a lamellar phase (*L*). The diffractograms of lamellar phases show a set of Bragg reflections whose reciprocal spacings are in characteristic ratios of Qn = 2πn/*d* (reflection order number n = 1, 2, 3. . .). The repeat distance d was obtained from the slope of the regression function of the dependence Qn = a x n, according to the equation *d* = 2π/a.

### Fourier transform infrared spectroscopy

Infrared spectra were collected with a Nicolet 6700 spectrometer (Thermo Scientific) equipped with a single-reflection MIRacle ATR ZnSe crystal (PIKE Technologies, Madison, USA). A clamping mechanism with constant pressure was used. Lipid films were prepared for the XRD measurements and were transferred to the ATR crystal. SC sheets removed from the Franz diffusion cells were placed directly on the ATR crystal. The spectra were generated by the coaddition of 256 scans collected at a resolution of 2 cm^−1^ at the ambient temperature and humidity. The spectra were analyzed using Bruker OPUS software. The exact peak positions were determined from the second derivative spectra.

### Data treatment

Data are presented as the mean ± standard deviation (SD), and the number of replicates is given in the pertinent figure. Statistical significance was analyzed using one-way analysis of variance with Dunnett’s post hoc test or a two-way method analysis of variance with Tukey’s post hoc test. *P* < 0.05 was considered significant and is shown in the figures as follows: *P* < 0.05 (∗), *P* < 0.01 (∗∗), *P* < 0.001 (∗∗∗), *P* < 0.0001 (∗∗∗∗).

## Results

### Topical SPC and GS diminish barrier function in isolated human SC sheets

We first wanted to find out if GS or SPC delivered in SC would have any effect on the barrier. To deliver sufficient lysosphingolipids into the tissue, we used an ethanol/propylene glycol mixture that has been used previously to deliver lipids or lipophilic active substances to the skin barrier in an experimental setup (39, 40, 41). Indeed, HPTLC analysis showed that SPC and GS reached 16% and 23% of the SC Cers by weight, respectively.

**Fig. 2:**
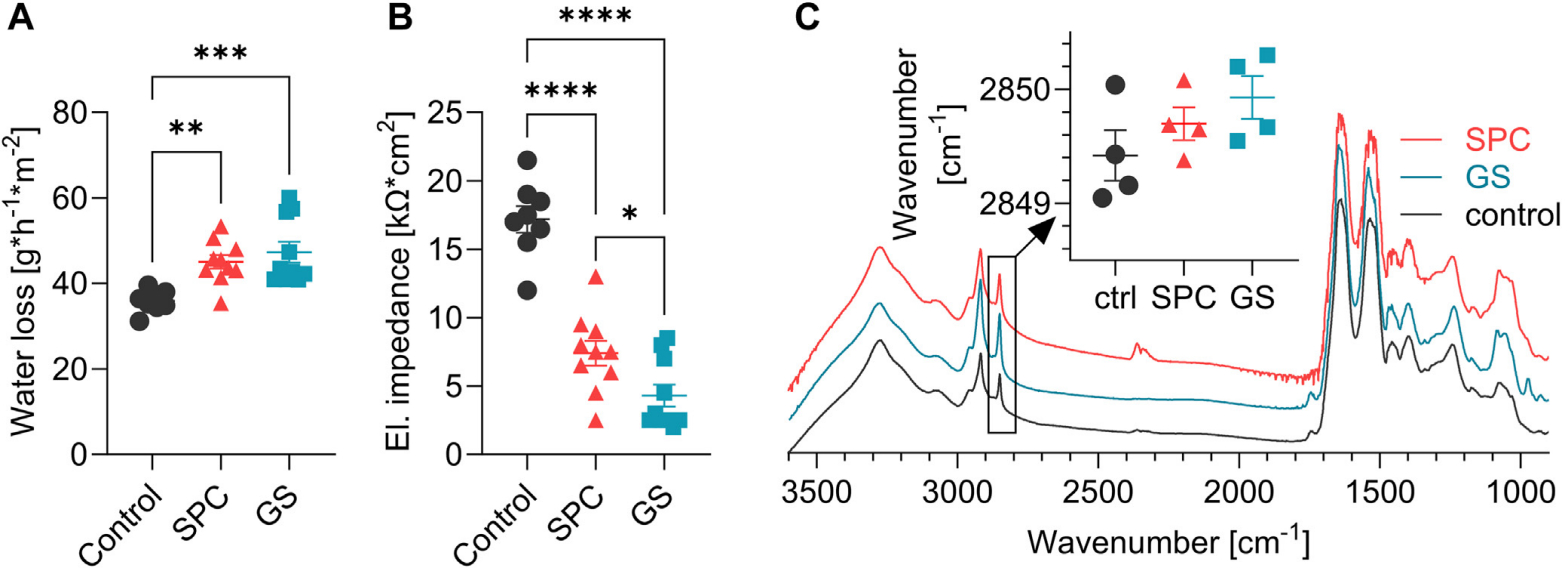
Effects of SPC and GS on isolated human SC sheets. Panel A: water loss, Panel B: electrical impedance, Panel C infrared spectra and SC lipid chain order estimated from the methylene symmetric stretching vibration (insert). Values are presented as individual values and means ± SD, n = 4–10. Asterisks indicate statistically significant differences as indicated (one-way Anova).

At these concentrations, SPC and GS increased water loss by 25% and 32%, and decreased electrical impedance 2- and 4-fold, respectively, compared to the solvent-treated control SC (Fig. 2). Infrared spectroscopy revealed slightly (but not significantly) disordered lipid chains in lysosphingolipid-treated SC compared to control, although the wavenumbers of the methylene symmetric vibration mostly remained below 2850 cm^−1^, indicating that well-ordered *all-trans* chains still dominated.

These results suggest that lysosphingolipids do indeed permeabilize SC lipids, although in this experimental setup, we only added the lysosphingolipids to SC sheets in vitro, using a mixture of solvents and such findings cannot be extrapolated to the clinical context.

### The presence of lysosphingolipids and additional FFAs does not adversely affect the lamellar phases of SC lipids, in contrast to a decrease in the Cer fraction below 80%

**Fig. 3:**
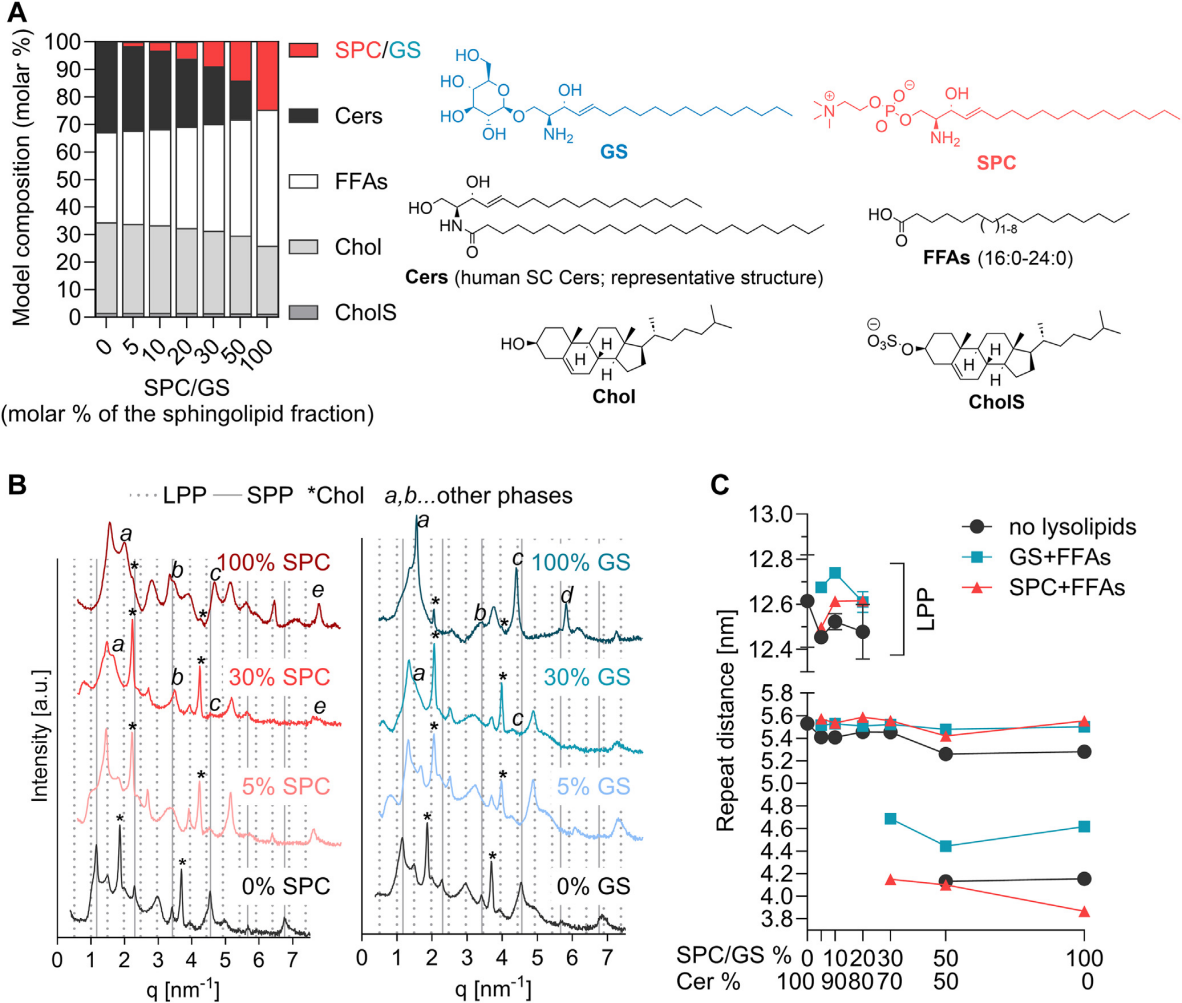
Composition and lamellar organisation of the model lipid films based on human SC Cers. Panel A: model composition in molar %. Cer refer to isolated human SC Cers. Panel B: Representative diffractograms of lipid mixtures; y-axis has a log10 scale; dotted vertical lines indicate LPP reflections, full vertical lines indicate SPP reflections, asterisks mark Chol reflections and letters mark additional phases. Panel C: lamellar repeat distances found in the studied lipid models; data are shown as means and SD (some SDs are smaller than the symbols), n ≥ 2.

To better understand the changes resulting from the gradual replacement of Cer by GS + FFAs or SPC + FFAs, we created lipid models in which we could precisely control the lipid composition. A model of a 'healthy' lipid barrier was prepared containing the following components: (i) a Cer fraction which was isolated and purified from human SC, (ii) a mixture of synthetic FFAs (16:0–24:0), (iii) Chol and (iv) CholS in a molar ratio of 1:1:1:0.13. This composition mimics the ratio of the major classes of barrier lipids in SC. The Cer composition found by HPTLC was 4.5% CerEO(d)S, 12.9% CerN(d)S, 25.5% CerNP, 2.9% CerEOH, 28% CerA(d)S/NH, 16.2% CerAP, 10% CerAH. Three series of models were then prepared: a first (control) series in which the amount of Cers was gradually reduced, a second series in which the amount of Cers was reduced in the same way and replaced by equal molar amounts of SPC and FFAs, and a third series in which the amount of Cers in the model was again reduced and replaced by equal molar amounts of GC and FFAs (Fig. 3A).

The X-ray diffractograms of the control lipid models, composed of human SC Cers/FFAs/Chol/CholS, showed three sets of reflections assigned to lamellae with a repeat distance of 12.6 ± 0.20 nm, corresponding to a long periodicity phase (LPP), the second phase with *d* = 5.5 ± 0.04 nm, assigned to a short periodicity phase (SPP) and separated Chol (*d* = 3.4 nm) (Fig. 3B, C). A reduction of up to 20% in Cers did not significantly affect the observed phases, although the intensity of LPP reflections faded and some were present only as shoulders of reflections belonging to SPP. In the model with a 30% reduction in Cer abundance, LPP was no longer observed, only SPP (or lamellar phases with SPP-like periodicity) and Chol was present. In the models containing only half of the Cer fraction or no Cers, phase separation occurred and two new phases with repeat distances of 5.3 nm (likely very long chain FFAs rich phase) and 4.1 nm were visible in the diffractograms, as well as Chol.

Gradual replacement of the human SC Cer fraction with SPC + FFAs or GS + FFAs up to 20% had no significant effect on the observed repeat distances; the LPP repeat distance varied between 12.5 and 12.7 nm and the SPP repeat distance was about 5.5 nm. At 30% Cers replacement, no LPP was detectable, and new shorter phases formed with repeat distances of 4.7 nm and 4.2 nm in GS- and SPC-containing models, respectively. No reflections corresponding to SPC or GS were observed in the diffractograms, indicating that there was no phase separation of these components from the mixture. This scenario persisted until the complete replacement of Cers by lysosphingolipids + FFAs. In the 100% SPC model, the repeat distance of the 4.2 nm phase decreased to 3.8 nm, which is very close to pure SPC (*d* = 3.9 nm). No separate GS (*d* = 3.5 nm) was observed in the diffractograms. It is noteworthy that the Chol reflections were weaker compared to the other reflections in these models, indicating an increased miscibility of Chol with (some of) these phases.

### SPC, but not GS, slightly disorders lipid chains

**Fig. 4:**
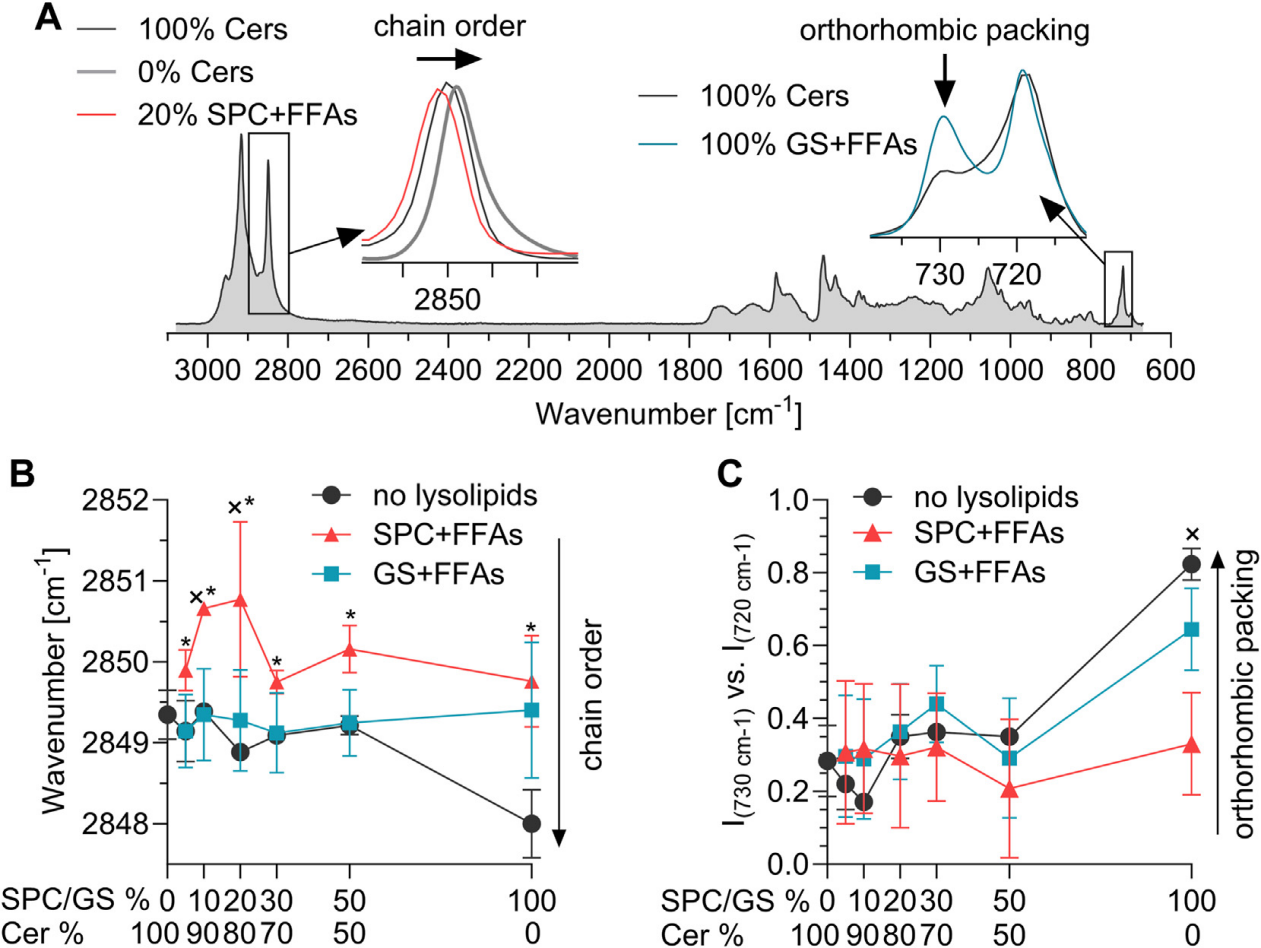
Infrared spectra, chain order and packing of the model lipid films based on human SC Cers. Panel A: Infrared spectrum of the control lipid film with inserts showing examples of methylene symmetric stretching vibration at about 2850 cm^−1^, indicative of chain order, and methylene rocking doublet at about 730 and 720 cm^−1^, sensitive to orthorhombic packing. Panel B: Wavenumbers of methylene symmetric stretching vibrations sensitive to lipid chain order. Panel C: Ratio of intensities of the 730 and 720 cm^−1^ components of the rocking doublet, a rough estimate of the orthorhombic packing content in the sample. Data are presented as mean and SD, n = 3–6. × indicates a significant difference from the control model with 100% Cers, black ∗ indicates a significant difference from models with the same Cer content and no lysolipids (two-way ANOVA).

These models were then studied using infrared spectroscopy (Fig. 4). Methylene symmetrical stretching is a good indicator of lipid chain order; well-ordered chains with a predominant all-*trans* conformation have wavenumbers below 2850 cm^−1^, which we observed in the control model (2849.3 ± 0.3 cm^−1^; Fig. 4A, B). Reducing human SC Cers by up to 50% did not significantly affect chain order in the model, only the model without human SC Cers had a lower wavenumber of this vibration, consistent with the observed separation of a phase probably rich in very long chain FFAs. Replacement of Cers by GS + FFAs did not affect the overall chain order in the models. In the case of SPC, this band shifted to higher wavenumbers, implying more *gauche* conformers in the lipid chains (especially in samples with 10%–20% SPC) compared to the model without lysosphingolipids.

The methylene rocking band is sensitive to lipid chain packing and the presence of a doublet at around 720 and 730 cm^−1^ (particularly its 730 cm^−1^ component) indicates the presence of very tight orthorhombic chain packing (Fig. 4A, C). The intensities of the doublet component at 730 cm^−1^ relative to the band at 720 cm^−1^ suggest an approximate representation of the orthorhombic phase in the sample. Fig. 4C shows that although the loss of part of the Cer fraction or its replacement by lysosphingolipids + FFAs leads to small fluctuations in the amount of orthorhombic phase estimated in this way, the changes are not significant. Only in samples with complete loss of Cers is an increase in orthorhombic chains observed, which may seem illogical at first sight, but it is related to the phase separation of FFAs in this sample.

### Lysosphigolipids + FFAs do not worsen the barrier function of the lipid models, but rather partially mitigate the loss of Cers

To investigate the effects of human SC Cer replacement by lysosphingolipids + FFAs on the functional properties of the models, the lipid films were sandwiched in Franz diffusion cells and water loss, electrical impedance, and permeability to two model permeants, TH and IND, were measured.

**Fig. 5:**
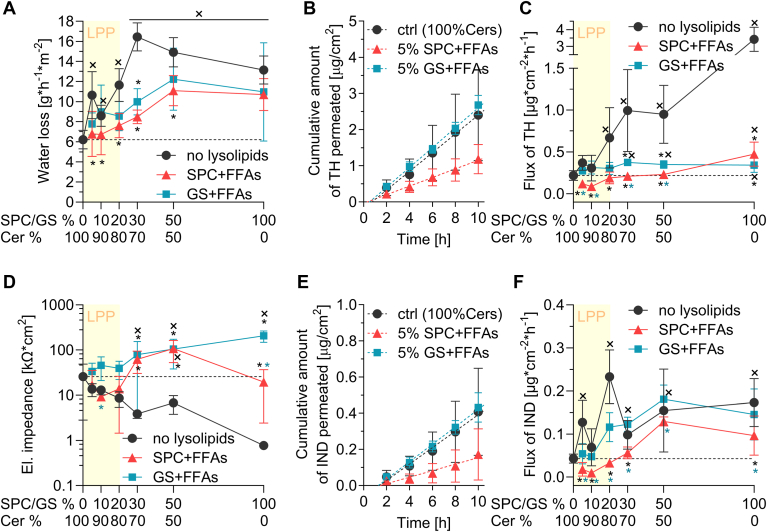
Permeability of the model lipid films based on human SC Cers for water, ions, TH, and IND. Panel A: Water loss through the lipid films. Panel B: Example permeation profiles for TH. Panel C: TH flux values. Panel D: Electrical impedance; y-axis has a log10 scale. Panel E: Example permeation profiles for IND. Panel F: IND flux values. Data are presented as mean and SD, n = 5. × indicates a significant difference from the control model with 100% Cers, and black ∗ indicates a significant difference from models with the same Cer content and no lysolipids. Blue ∗ indicates a significant difference against models with same Cer content and GS + FFAs (two-way ANOVA).

The water loss of the control human SC Cers/FFAs/Chol/CholS model was found to be 6.2 ± 0.9 g∗h^−1^∗m^−2^ (Fig. 5A). Replacement of the Cer fraction with SPC/FFAs or GS/FFAs resulted in a gradual deterioration of the water barrier. Water loss values were not significantly different from the control up to 20% Cer replacement, whereas 50%–100% Cer replacement resulted in almost two times higher water loss values compared to the control (up to ∼12 g∗h^−1^∗m^−2^). No differences were observed between SPC and GS. However, this increase in water loss is clearly not due to the presence of SPC or GS, but to the reduced amount of Cers in the models, as clearly seen in the samples where only Cers were reduced without replacing it with lysosphingolipids + FFAs, which resulted in up to a 2.5-fold increase in TEWL (up to ∼16 g∗h^−1^∗m^−2^). Fig. 5A shows that replacing the missing Cers with the same molar amount of lysosphingolipids and FFAs leads to a decrease in the model permeability to water.

Electrical impedance is negatively related to ion permeation - the decrease in values after removing some or all of the Cers from the model seen in Fig. 5D (from ∼26 kΩ∗cm^2^ in control to ∼1 kΩ∗cm^2^ in the sample without Cers) therefore indicates a lower barrier to ions. In contrast, replacing the missing Cers with GS + FFAs resulted in higher impedance values (up to∼200 kΩ∗cm^2^), especially in samples where LPP was no longer evident (for illustration, the presence of LPP is indicated by yellow rectangles). Replacement of Cers with a mixture of SPC + FFAs initially (in the models where LPP still formed) led to a decrease in impedance to values comparable to the lysosphingolipid-free samples with the same Cer levels, but with 30% replacement of Cers with SPC + FFAs, impedance increased dramatically to values comparable to 30% GS + FFAs. The same scenario was observed for samples with 50% replacement of Cers with lysosphingolipids. In models where all Cers were replaced by SPC + FFAs, the impedance values decreased again to values comparable to the control with 100% Cer fraction.

The flux of TH through the control models was 0.22 ± 0.06 μg∗cm^−2^∗h^−1^ (Fig. 5B, C) and increased significantly when Cers were removed from the model (especially when LPP was lost). Surprisingly, the replacement of Cers by GS + FFAs prevented the increase in permeability for TH - values remained around 0.3 μg∗cm^−2^∗h^−1^ even in samples where Cers were completely replaced by GS + FFAs. The effect of SPC + FFAs was even stronger. When 5%–10% of the Cer fraction was replaced by SPC + FFAs, TH flux actually decreased to values less than half of the control (illustrated by the permeation profiles in Fig. 5B). With further replacement of Cers by SPC + FFAs (up to 50%), TH flux was comparable to the control with 100% Cers, only in models with full replacement of Cers by SPC + FFAs, TH flux was more than double that of the control.

The flux of our second model permeant, IND, was 0.04 ± 0.01 μg∗cm^−2^∗h^−1^ and the gradual removal of Cers from the model resulted in a fivefold increase in its permeation rates compared to the control (Fig. 5E, F). Given the variability in flux values, we can say that the permeability for IND for samples containing GS + FFAs was similar to that for samples where only the Cer fraction was reduced. The effects of SPC on the permeability to IND were significantly different from those of GS. SPC + FFAs at 5%–10% actually reduced the flux of IND by 2.5–4 times, as shown by the permeation profiles in Fig. 5E. 20% SPC + FFAs resulted in an IND flux similar to that of the 100% Cers control, and at higher SPC + FFAs levels the permeability to IND reached values roughly comparable to those of the controls without lysosphingolipids.

## Discussion

Hydrolysis of Cer precursors to lysosphingolipids GS or SPC and FFAs by the enzyme SM-GlcCer-deacylase has been found in the skin barrier of patients with atopic dermatitis (42, 43). Since this deacylation is one of the factors of Cers deficiency in the SC extracellular lipid matrix, it has been suggested that the presence of this enzyme is one of the important factors contributing to the poor barrier function observed in these patients. Depending on the severity of the disease, these patients have up to a 3-fold higher TEWL (28, 44, 45, 46) and a 2- to 3.5-fold higher permeability to exogenous substances (47) than healthy volunteers. Among the structural parameters of the lipid matrix, a lower proportion of LPP (48), less ordered lipid chains (46), and less orthorhombic chain packing (46) were observed in atopic dermatitis patients compared to healthy individuals, parameters that correlate with barrier function.

Although it seems intuitive that the substitution of a Cer for a lysosphingolipid and a FFA in the SC lipid matrix would affect barrier structure and function, this cannot be stated unequivocally from the clinical data. There are a number of other changes in the lipid profile in this disease [13] (eg insufficient acyl elongation (49), changes in the ratio of individual subclasses of Cers, eg less ultra-long ω-*O*-acylCers such as CerEOS, which is associated with reduced LPP (45)). Our pilot data, where we simply applied a 1% solution of lysosphingolipids (GS or SPC) to human SC sheets, suggested that lysosphingolipids have a negative effect on skin barrier function as estimated by water loss and electrical impedance, and slightly disorder SC lipid chains.

We therefore decided to create lipid models in which this pathological pathway from GlcCers or SMs to lysosphingolipids and FFAs (instead of Cers) could be studied isolated from other contributing factors. As we wanted to gradually replace Cers with a mixture of lysosphingolipids and FFAs in the models, it was not possible to use complete human SC lipids, but it was necessary to isolate the Cer fraction, similar to what we have described in previous studies (32, 50). In the control sample, we added a mixture of FFAs, Chol, and CholS to this human SC Cer fraction in proportions corresponding to their ratios in SC. These control samples mimic very well important structural aspects of SC lipids: the coexistence of LPP, SPP and separated Chol (51), the dominance of well-ordered chains, and the presence of orthorhombically packed domains. Previous studies suggest that more than 5.4 M % of ω-*O*-acylCers are required for LPP formation (LPP formed at 7% acylCers (52) or 7.5% acylCers but not at 5% (53) in lipid models, but not in atopic patients with only 5.4% acylCers (54, 55)). This is in agreement with our samples, where about 7.4% Cers belonged to the ω-*O*-acylCer subclasses.

In these samples, we then replaced a certain percentage of the human SC Cer fraction with a mixture of lysosphingolipids and FFAs. It should be noted that, due to commercial unavailability, neither the lysosphingolipids used (which have only sphingosine with 18 carbons as a backbone) nor the mixture of FFAs (which do not contain ultra-long chains) reflect the full structural complexity of the compounds as one would expect from the deacylation of SMs and especially GlcCers in the epidermis. Models in which Cers were similarly reduced without replacement by lysosphingolipid + FFAs were prepared as further controls.

Loss of 30% or more Cers resulted in increased permeability for all markers studied. Given that a ≥30% reduction of Cers results in an analogous decrease in ω-*O*-acylCer (e.g. CerEOS), one would expect that an important structural element of the lipid skin barrier, LPP, would be greatly reduced or disappear in these models, and this was indeed observed. With 50% or no Cers in the model, even SPP separated into two phases, consistent with the low miscibility of Chol with very long chain FFAs. This separation of FFAs (likely those with 22:0–24:0 chains) is consistent with the higher lipid chain order and more orthorhombically packed chains observed in these samples. Phase separation can also explain the increased permeability of these lipid films.

When we replaced the reduced human SC Cers with an equal molar amount of GS + FFAs or SPC + FFAs, no further permeabilization was observed. On the contrary, the lysosphingolipids (especially SPC) with FFAs partially mitigated the negative effects of the lack of Cers. The increases in water loss and TH flux were lower and the effects on IND permeation were similar or less pronounced than those seen without lysosphingolipids. Electrical impedance actually indicated a stronger barrier to ion movement, probably related to the ionic/ionizable nature of lysosphingolipids and FFAs, which can alter this barrier measure. XRD of these samples indicated that SPP (or a lamellar phase with SPP-like periodicity) was still present in these samples, although another phase separated from it, similar to the samples without lysosphingolipids. Notably, the intensity of the separated Chol reflection(s) was relatively reduced, indicating its increased miscibility with one of these newly formed phases. Since Chol does not mix with very long FFAs (56, 57), it is likely that some of the newly formed phases are rich in Chol and lysosphingolipids (whose 18C hydrophobic chain matches Chol), except for 100% SPC where the repeat distance of the separated phase indicated almost pure SPC. The persistence of lamellar phases with SPP-like periodicity in samples with 50% or no Cers is intriguing and suggests that the lysosphingolipid, possibly as an ion pair with FFA, may partly mimic the structural arrangement of Cer in such phase (see below).

Although the above models, in which a substantial fraction or all the human SC Cers are removed/replaced, are important for understanding the potential magnitude of lysosphingolipid effects, models with lower levels of lysosphingolipids are much closer to the pathophysiological situation. Unfortunately, we do not know the exact concentrations of lysosphingolipids in SCs. Studies quantifying SPC or GS in SCs from atopic patients report tens of ng of lysosphingolipids per mg of SC (14, 42). However, these data are not consistent with the decrease in Cer concentrations (in the order of μg/mg, (28)). Possible explanations could be that these data come from the three uppermost tape strips where lysosphingolipids were depleted or degraded, or that the strips were extracted with very non-polar solvents that do not dissolve lysosphingolipids well. It is therefore possible that these data represent only a fraction of the local lysosphingolipid concentration in the deeper SC layers, which is uncertain. Therefore, in this work, we further prepared models in which we successively replaced 5%, 10% and 20% Cer fractions with the deacylation products of their precursors, ie a mixture of lysosphingolipids and FFAs, to better describe the effect of these molecules on the structure and function of the lipid skin barrier and to investigate the hypothesis that even small amounts of these lysosphingolipids have a negative effect on the barrier.

Replacing 5 or 10 mol% of the human SC Cer fraction in these models with a mixture of lysosphingolipid (GS or SPC) and FFAs gave rather surprising results - the barrier was not significantly disrupted and the presence of SPC even led to a reduced permeability of the lipid matrix. LPP was still present in these models, although its reflections were less pronounced and the diffractograms were dominated by SPP. In the case of SPC, the lipid mixture tolerated even a 20% replacement of the Cer fraction by SPC + FFAs without a significant increase in permeability.

The effects of 5%–10% SPC + FFAs, causing an apparent barrier strengthening to model exogenous permeants TH and IND, are intriguing. Unlike the glucose residue in GS, the phosphocholine in SPC is zwitterionic, offering the possibility of ionic interactions with the permeants. However, such an effect would be expected to become more pronounced with more SPC in the model, which we did not observe. Infrared spectroscopy showed that the lipid chains in the models with 10% SPC are in fact less ordered than their counterparts with 10% less Cers. Whether these less-ordered lipid chains are in LPP or SPP cannot be deduced from these experiments. Alternatively, these slightly more fluid lipids may be separated from these phases because XRD only detects ordered and periodically repeating structural elements. The less ordered lipids seem counterintuitive as one would expect less permeable lipid films to have more ordered lipids. A possible explanation is that these lipids may reduce some putative permeability defects between the LPP and SPP, as this barrier reinforcing effect of SPC + FFAs disappears with the loss of LPP.

It can be concluded that the partial hydrolysis of GlcCers and SMs to lysosphingolipids + FFAs does not significantly disturb the structure or function of the lipid skin barrier as long as the amount of human SC Cers, including ω-*O*-acylcers, is not reduced to the point where it would not allow lipid assembly into LPP. This result seems to be different from the effect of lysophospholipids in phospholipid bilayers, where as little as 1% lysophospholipid destabilize and permeabilize membranes (22, 58, 59). However, it is important to be aware of the different structure of SC lipids and the environment in which they are found: unlike phospholipids, which are cylindrical and readily form bilayers surrounded by an aqueous environment, SC extracellular lipids are stacked in multilayered formations, interconnected by Cers in an open conformation (also called extended or splayed-chain conformation, ie with chains pointing in opposite directions). This is probably a way of minimising the packing stress of these molecules with small polar heads, which in the hairpin conformation would be inverted cone-shaped and prefer negative curvature. SC lipids are also minimally hydrated. Another possible explanation for the discrepancy between the destabilising effects of lysophospholipids reported in the literature (22, 58, 59) and our findings is the difference in curvature, as the literature data were predominantly obtained from vesicles, where curvature plays a significant role. In our work, however, a direct comparison with these systems was neither intended nor technically feasible, as SC lipids form vesicles only at high pH, which would introduce undesirable effects into the study.

Lysolipids are basically surfactants with a large charged polar head and a single hydrophobic chain, ie cone-shaped lipids with a preference for positive curvature. Unlike Cers, they are also much more soluble in water and form micelles. These two features of lysolipids then destabilize planar phospholipid membranes. In the case of SC lipid multilayers, however, the substitution of Cer by lysosphingolipid + FFA is not a substitution of cylindrical by conical lipid, but a substitution of splayed-chain lipid by two single-chain lipids, which may still be held together by ionic bonds given the minimal water content of this layer.

In conclusion, the pathological breakdown of GlcCers and SMs by the enzyme SM-GlcCer-deacylase into lysosphingolipids (SPC or GS) and FFAs, instead of the physiological metabolism into Cers, as observed in patients with atopic dermatitis (12, 13), negatively affect the structural and functional properties of skin barrier lipids. However, these negative effects are related to the reduced levels of Cers (and consequently the loss of LPP) and not to the direct deleterious effect of the mixture of lysosphingolipids and FFAs, as these components can compensate to some extent for the loss of Cers. Furthermore, these findings raise new questions regarding the behavior of lysolipids in rigid multilamellar lipid environments, such as the extracellular lipid matrix of the SC, which may differ significantly from their behavior in phospholipid bilayers. This is particularly relevant for the physiological conversion of phospholipids extruded from lamellar bodies into FFAs (a component of the lipid barrier) and lyso-phospholipids, the fate of which in the epidermis remains incompletely understood.

## Data availability

Data are available from the authors upon request.

## Conflict of interests

The authors declare that they have no conflicts of interest with the contents of this article.
